# Evaluation of Fixation Disparity Curve Parameters With the Modified Near Mallett Unit in Symptomatic and Asymptomatic University Students

**DOI:** 10.5812/ircmj.8572

**Published:** 2013-11-05

**Authors:** Hamed Momeni Moghadam, David A Goss, Abbas A Yekta, Marzieh Ehsani

**Affiliations:** 1 Health Promotion Research Center, Zahedan University of Medical Sciences, Zahedan, IR Iran; 2 School of Optometry, Indiana University, Bloomington, Indiana, USA; 3 School of Paramedical Sciences, Mashad University of Medical Sciences, Mashhad, IR Iran; 4 School of Rehabilitation Sciences, Zahedan University of Medical Sciences, Zahedan, IR Iran

**Keywords:** Fixation Disparity, Visual Symptoms, Binocular Vision

## Abstract

**Background:**

Research suggests that fixation disparity data are extremely useful in the assessment of the binocular and accommodative systems.

**Objectives:**

The purpose of this study was to evaluate fixation disparity curve (FDC) parameters with a modified near Mallett unit in symptomatic and asymptomatic students of Paramedical Sciences School of Mashhad University of Medical Sciences in 2007.

**Patients and Methods:**

In this analytical-descriptive study, 100 students were selected randomly and divided into symptomatic and asymptomatic groups. Fixation disparity curve parameters were determined for each subject and compared in symptomatic and asymptomatic groups.

**Results:**

There were more subjects with exo fixation disparity than eso fixation disparity in the study sample. The means for fixation disparity, fixation disparity curve x-intercept, and slope with the modified Mallett unit were each significantly different by Mann-Whitney U test in the symptomatic and asymptomatic groups. Also there was a significant difference in the distributions of fixation disparity curve types in the two symptom groups by Chi-square test.

**Conclusions:**

The X-intercepts (point at which the FDC crosses the X-axis) were on average more in the base-in direction, Y-intercepts (point at which the FDC crosses the Y-axis) were shifted in the exo direction, and slopes were steeper in the symptomatic group.

## 1. Background

Fixation disparity is a small misalignment of visual axes in the presence of binocular alignment (1) within the limitations of Panum’s fusional space (2). The misalignment (a few minutes of arc) is much smaller than that of strabismus. It has been suggested that fixation fisparity (FD) has the potential to provide a more real status of binocular system function than other systems of binocular analysis (3). Studies have shown that FD can be an indicator of vergence accuracy under binocular conditions and a sign of binocular stress (4-6). Evaluation of FD gives the examiner information that can be used in the planning of treatment options with lenses, prism and/or vision training techniques (7-10).

A fixation disparity curve (FDC) is an X-Y plot that shows change in FD with varying amounts of relative vergence effort induced by application of prism (11). FDC parameters which are useful in analysis of binocular vision anomalies include: X-intercept, Y-intercept, curve shape, center of symmetry, curve slope (12). Fixation disparity in minutes of arc with zero prism in place is the point at which the FDC crosses the Y-axis (Y-intercept). The X-intercept is point on the curve where fixation disparity is zero. For determination of curve slope, attention is usually given to the change in FD between 3 prism diopters base in and 3 prism diopters base out (12).

Curve type or shape is another FDC parameter. The type of curve is usually determined based on Ogle’s classification of curves (13, 14).

Curve type 1 has a sigmoid shape with a flatter central portion and a steeper peripheral portion in both BI and BO directions. It has the best prognosis for the patient. Type 2 is often seen in symptomatic esophoric patients and type 3 typically in exophoric patients. These type 2 and type 3 curves typically have flatter portions, with the flat portion being mostly on the BO side in type 2 curves and mostly on the BI side in type 3 curves. Ogle has reported the prevalence of curves type 1, 2, 3, 4 as 60%, 25%, 10% and 5%, respectively (13). FDC type has diagnostic value in determination of vergence anomalies and can be important during the treatment process (15). The center of symmetry is a region in the center of the curve where curve slope is least due to rapid adaptation to vergence changes (14). Griffin and Grisham stated that abnormal FDCs have characteristics which include: significant amount of FD in zero prism position, high amount of X-intercept, steep curve (more than 45° related to Y-axis), and limited fusional vergence ranges (4).

The commercially available Mallett unit measures only the amount of prism needed to reduce fixation disparity to zero, but it can be modified to measure amount of fixation disparity. In the modified Mallett fixation disparity unit, the OXO lettering acts as a central fusion lock and the two green polarized nonius lines exactly above and below the center of X are used for FD measurement. There is an important difference between this device and the original Mallett unit. With the original one, we can measure only associated phoria, but in the modified instrument we are able to determine FD in minute arc. Accuracy of measurement with this device has been reported to be 0.25 minute arc (16, 17). The paragraph on the screen which acts as peripheral fusion lock was in Persian for use with Iranian persons in this study.

Because there may be a high prevalence of binocular vision anomalies among groups of school and university students (18), examination for binocular vision conditions is important. Sheedy and Saladin (7) have shown that FD is related to symptoms, so fixation disparity evaluation may be a helpful test to supplement measurement of heterophoria and vergence ranges, and it may provide a useful reflection of subject performance in the real world, particularly at near fixation distances.

## 2. Objectives

The purpose of this study was to examine FDC parameters with the modified Mallett fixation disparity unit in university students and to examine differences in the parameters between symptomatic and asymptomatic subjects.

## 3. Patients and Methods

In this analytical-descriptive study, 100 Iranian students of paramedical sciences school of Mashhad University of Medical Sciences were randomly selected from the list of students. This study was approved by research committee of Mashhad University of Medical Sciences (Code: 85306, Date: 23.08.2007).

We introduced the project to the invited students and gave any necessary explanations about the study. In addition, we assured subjects that their information was kept confidential in accordance with the tenets of the declaration of Helsinki. If they met inclusion criteria and consented, they were entered into the study. Then subjects were divided into two groups (symptomatic and asymptomatic) according to the convergence insufficiency symptom survey questionnaire (19). Subjects with a survey score 21 or greater were considered to be symptomatic.

Inclusion criteria and exclusion criteria helped to exclude subjects who may have had symptoms that were not due to vergence problems. Inclusion criteria were visual acuity 20/20 or better in each eye at 6m and 40cm with or without correction, absence of strabismus at 6m and 40cm with cover test, and no history of ocular trauma or ocular disease.

Refractive errors were determined by retinoscopy (Heine β-200 retinoscope). Cycloplegic retinoscopy was performed if any of the following were found: esophoria especially associated with a slow or jerky recovery movement on cover test, unstable objective or subjective refraction, large discrepancy between objective and subjective results, or spasm of the near triad (2). The results of retinoscopy were refined by subjective refraction, and finally dissociated red-green balance test was performed. Subjects used their corrections at least four weeks. After this, we used plate four of the TNO test to rule out suppression.

**Figure 1.**
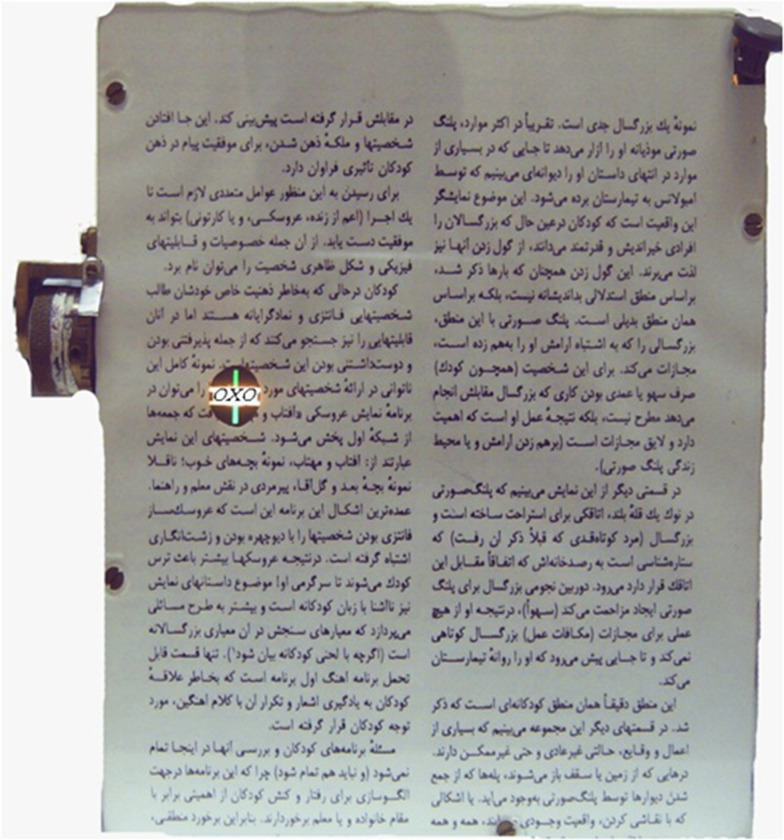
Modified Mallett Near Unit

FDC parameters were determined with the modified near Mallett unit (Figure 1) in each subject. 

For measurement of FD with the modified unit, the device was placed at 40cm from subjects. For subjects who had changes in their refractive errors from their habitual corrections, the corrective lenses were placed in a trial frame during testing. They wore polarizing spectacles and read four sentences from printed paragraphs on the screen to encourage stability of accommodation and convergence at this specific distance.

The presence and direction of FD were determined from the displacement of nonius lines relative to the center of the X and the direction of displacement. With the use of an adjustable drum, the position of the lines was adjusted until the two lines were reported by the subjects to be in line with the center of the X using the bracketing technique. The number on the drum was read and recorded as FD in minutes of arc. FD was determined with prism steps of 4 prism diopter in both horizontal directions was determined until the breakdown of fusion (17). First, a prism bar with base-in (BI) prism was placed in front of one eye of the subjects and the amounts of FD with increasing prism powers were determined. Then subjects had a 10 minute rest and measurements with base-out (BO) prisms were determined (20). For prevention of prism adaptation, prisms were not in place in front of the eyes for more than 15 seconds (21).

After plotting the FDCs, they were compared with Ogle’s curves, subjectively, for determination of curve type. Curve slope was calculated with changes of FD between 4 prism diopters base-out and 4 prism diopters base-in prisms divided by 8. It is more common to use 3 prism diopter BI and 3 prism diopter BO points to determine slope, but the prism bar used in this study did not have a 3 prism diopter prism. To reduce some potential biases, one examiner asked the subjects the questions about symptoms and did preliminary evaluations, and another examiner measured FD.

Near dissociated heterophoria was determined with alternate cover test method with best correction in trial frame and with subjects fixating on an accommodative target which was a small isolated letter "E" of approximately 20/30 (6/9) on a metal rod at eye level at 40 cm. As the alternate cover test was performed, the prism power was adjusted until there were no recovery movements in either eye. For confirmation of the neutral point, the prism power was increased until a reversal movement was seen. Then power was reduced until no movement was seen. The final results were cross checked with the subject’s response using a subjective Chi test.

For measurement of stereopsis with the TNO test, the red and green anaglyphic filters were worn and the booklet was held at 40 cm perpendicular to the subject’s visual axis. At first, the screening plates (plates I, II, III, IV) were shown. If these were successfully completed, the graded plates from 480 to 15 seconds of arc were presented until the subject was unable to identify the three-dimensional shape correctly. The lowest disparity that the subject was able to detect was recorded as his/her stereoacuity in seconds of arc.

After data collection, data were analyzed in SPSS version 15. Data were assessed for normality with the Kolmogorov-Smirnov test which did not indicated normal distribution permitting the use of non-parametric statistics. The Mann-Whitney U and Chi-square tests were used for analysis.

In all tests, the significance level was considered to be 0.05. In the calculations, exo fixation disparity and base-in X-intercept were negative numbers. Eso fixation disparity and base-out X-intercept were positive numbers.

## 4. Results

Of the 100 students under study, 53 (53.0%) were female and 47 (47.0%) male. The mean ages for all subjects and separately in females and males were 22.8 ± 2.3, 23.1 ± 2.1, 21.6 ± 9.1 years, respectively. Symptomatic and asymptomatic subjects numbered 30 subjects (30.0%) and 70 subjects (70.0%), respectively. The mean score of CISS for the symptomatic and asymptomatic group was 35.8 ± 10.2 and 13.3 ± 6.5. The Mann-Whitney U test showed a considerable difference in the mean score between the two groups (P < 0.001). The mean age, stereopsis, and dissociated heterophoria for all subjects and separately in the two symptom groups are presented in Table 1. 

**Table 1.** Mean ± SD for Age, Stereopsis and Dissociated Heterophoria ^a ^

Variables	All, Mean (SD), (95% CI) (n = 100)	Symptomatic, Mean (SD), (n = 30)	Asymptomatic, Mean (SD), (n = 70)	P value
**Age, y**	22.79 ± 2.35 (22.32 to 23.26)	22.57 ± 2.52 (21.62 to 23.51)	22.89 ± 2.29 (22.34 to 23.43)	0.53
**Stereopsis, sec. arc**	37.05 ± 17.11 (33.65 to 40.45)	52.0 ± 13.49 (46.96 to 57.04)	30.64 ± 14.31 (27.23 to 34.06)	< 0.001
**Dissociated phoria, prism diopters**	- 4.64 ± 5.69 (-3.51 to -5.76)	- 7.16 ± 7.68 (-4.29 to -10.03)	- 3.55 ± 4.21 (-2.55 to -4.56)	0.003

^a^The Mann-Whitney U test showed significant differences in mean stereopsis and dissociated phoria between the two groups but not in age. The mean and standard deviation of three FDC parameters (X-intercept, Y-intercept, and slope (4 BI to 4 BO slope) with the modified Mallett FD unit are displayed in Table 2.

**Table 2.** Mean ± SD for associated Phoria, Fixation Disparity and Curve Slope in All Subjects and Separately in Symptomatic and Asymptomatic Groups ^a ^

FDC Parameters	All, Mean (SD) (95% CI), (n = 100)	Symptomatic, Mean (SD), (95% CI) (n = 30)	Asymptomatic, Mean (SD), (95% CI) (n = 70)	P value
**X-Intercept**	- 0.61 ± 1.5 (- 0.91 to - 0.31)	- 1.35 ± 2.2 (- 2.20 to - 0.49)	- 0.3 ± 0.9 (- 0.51 to - 0.08)	< 0.001
**Y-Intercept**	- 0.51 ± 1.1 (- 0.73 to - 0.29)	- 1.09 ± 1.6 (- 1.70 to - 0.47)	- 0.26 ± 0.65 (- 0.42 to - 0.10)	< 0.001
**Curve Slope**	- 0.18 ± 0.2 (- 0.23 to - 0.14)	- 0.34 ± 0.3 ( -0.45 to - 0.23)	- 0.12 ± 0.10 (- 0.15 to - 0.09)	< 0.001

^a^ The mean X-intercept in the symptomatic group is more BI compared with the asymptomatic group, with a difference of 1.05 prism diopters between two groups. Also the mean Y-intercept in the symptomatic group is more exo-FD compared with asymptomatic group, with a mean difference of 0.83 minutes of arc. In addition, the average curve slope in the symptomatic group is 0.22 minutes of arc/prism diopter steeper than in the asymptomatic group. The Mann-Whitney U test showed significant differences in mean X-intercept, Y-intercept, and curve slope between the two groups.

**Figure 2.**
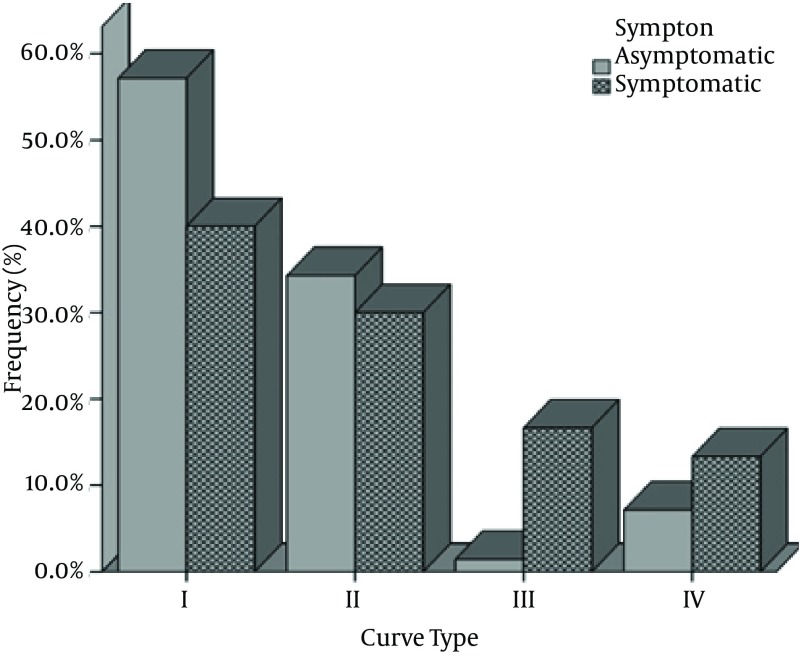
Ogle FD Curve Types by Symptom Group

Another FDC parameter is type of FDC. The distribution of frequency of different types of FD curves with the modified near Mallett unit is displayed in Table 3 and Figure 2. 

**Table 3.** Frequencies of FDC Types with the Modified Near Mallett Unit

Curve Type	Asymptomatic, No. (%)	Symptomatic, No. (%)	Total, No. (%)
**I**	40 (57.1)	12 (40.0)	52 (52.0)
**II**	24 (34.3)	9 (30.0)	33 (33.0)
**III**	1 (1.4)	5 (16.7)	6 (6.0)
**IV**	5 (7.1)	4 (13.3)	9 (9.0)
**Total**	70 (100.0)	30 (100.0)	100 (100.0)

With the modified Mallett FD unit the most prevalent type of curve was type I (53.0%), followed by type II (33.0%), type IV (9.0%), type III (6.0%). All curves could be classified into one of the four curve types. Type I curves were the most common in both groups, but type I curves were found in 57.1% of asymptomatic subjects and 40% of symptomatic subjects. Although the counts were small, it may be noted that only 8.5% of asymptomatic subjects had type 3 or 4 curves, compared to 30% of the symptomatic subjects. The χ^2^ test shows a statistically significant difference in the distribution of curve type with symptoms. (χ^2^ = 10.325, df = 3, P = 0.01)

Table 4 shows the distribution of direction of dissociated phoria from the cover test and fixation disparity. 

**Table 4.** The Distribution of Direction of Fixation Disparity In Relation to Direction of Dissociated Phoria

Group and Dissociated phoria	Exo FD, No. (%)	Zero FD, No. (%)	Eso FD, No. (%)	Total, No. (%)
**Asymptomatic**				
Exo	24 (34.28)	23 (32.85)	1 (1.43)	48 (68.56)
Ortho	1 (1.43)	15 (21.44)	1 (1.43)	17 (24.30)
Eso	0 (0.00)	0 (0.00)	5 (7.14)	5 (7.14)
Total	25 (35.81)	38 (54.39)	7 (10.00)	70 (100.00)
**Symptomatic**				
Exo	24 (80.00)	1 (3.33)	0 (0.00)	25 (83.33)
Ortho	0 (0.00)	0 (0.00)	0 (0.00)	0 (0.00)
Eso	0 (0.00)	0 (0.00)	5 (16.67)	5 (16.67)
Total	24 (80.00)	1 (3.33)	5 (16.67)	30 (100.00)

Most subjects (88) had either exo fixation disparity or zero fixation disparity. Only 12 subjects (12.0%) had eso fixation disparity. Only 10 subjects (10.0%) had an eso dissociated phoria. Among all subjects, there was only one case of paradoxical fixation disparity (eso fixation disparity associated with exophoria). Zero fixation disparity was observed in 38 of the 70 (54.4%) asymptomatic subjects, but only 1 of the 30 (3.3%) symptomatic subjects. Exo fixation disparity was found in 24 of the 48 (50.0%) asymptomatic subjects with exo dissociated phoria. In comparison, exo fixation disparity was found in 24 of the 25 (96.0%) symptomatic subjects with exo dissociated phoria.

## 5. Discussion

There were more subjects with exo dissociated phoria than eso dissociated phoria in this study. The findings of this study show a more divergent y-intercept, a more BI X-intercept, and a more negative slope in the binocular symptomatic subjects than in the asymptomatic subjects. However, the differences were fairly small. For example, the difference in mean X-intercept between groups was about one prism diopter, and the difference in mean Y-intercept between groups was less than one minute of arc. It could be argued that these are not clinically significant differences.

The results of this study are consistent with the results of Cornell et al.(22). This study showed that curve slope has diagnostic value, confirming the results of several other studies (7, 15, 23) . In the present study, the average curve slope in symptomatic and asymptomatic groups was -0.34 and -0.12 minutes of arc/prism diopter, respectively. In comparison, Sheedy and Saladin (7) reported if the central curve slope was flatter than -0.96 subjects tended to be asymptomatic but if was steeper, they were likely to be symptomatic. Their reported cut-off point was higher than our amounts. This difference may attributed to the fixation disparity devices used in the two studies and also ranges of prism powers used in the calculation of curve slope. They used the Sheedy disparometer which does not have central fusion lock, and curve slope was calculated from 3 prism diopters base-out to 3 prism diopters base-in (7, 15). In contrast, we used the modified near Mallett unit which does have a central fusion lock, and curve slope was determined between 4 prism diopters base-out and 4 prism diopters base-in. Curve slopes have been found to differ on different fixation disparity devices (24). Yekta et al. reported that an X-intercept of 1 prism diopter or more in BI direction in pre-presbyopes was likely to be associated with symptoms (17). The subjects in our study were also of pre-presbyopic age and the mean X-intercepts in the symptomatic and asymptomatic groups were -1.35 and -0.3 prism diopter, respectively.

Ogle et al. suggested that fixation disparity and curve slope are the most important FDC parameters (13). Also, Saladin and Carr reported that fixation disparity and curve slope are the best for differentiating asymptomatic from symptomatic subjects (25). Our findings show differences in each of three parameters of FDCs (Y-intercept, curve slope and X-intercept) in the symptomatic and asymptomatic groups. Sheedy found that Y-intercept, X-intercept, and curve slope in asymptomatic subjects were 3.5 minutes of arc exo-FD, 3.3 prism diopters BI, and 0.7 minutes of arc/prism diopter, respectively (15). In the present study the mean values of these variables were 0.26 minutes of arc exo-FD, 0.3 prism diopters BI X-intercept, and 0.12 minutes of arc/prism diopter, respectively. These differences may be related to the type of FD devices used in the Sheedy study (Sheedy disparometer) and in the present study (modified near Mallett unit). The lack of a central fusion lock in the Sheedy disparometer could lead to the higher FDC variables compared with the modified Mallett unit.

It appears that the results of the present study differ from those of Collier and Rosenfield (26). They found that in 20 young adults doing a thirty minute computer screen reading task those who had the lowest discomfort level had a mean associated phoria of 1.6 prism diopter BI and those who had the highest discomfort had a mean associated phoria of 0. However, they used an associated phoria target without a central fusion lock. In addition, it appears that there may have been more subjects with base-out associated phorias among the more symptomatic subjects, and they stated that they could not rule out other etiologies for computer use discomfort such as eye movement disorders or tear layer abnormalities (26). The results of the present study are more consistent with those of Yekta and Pickwell (27). They also used a modified Mallett unit to measure fixation disparity. For 36 symptomatic subjects in their study, there was a mean 1.56 minutes of arc exo fixation disparity and for 49 asymptomatic subjects, there was a mean 0.26 minutes of arc exo fixation disparity. In another study with a modified Mallett unit, Yekta et al. (17) reported average fixation disparity findings of 1.79 minutes of arc exo in symptomatic non-presbyopes and 0.29 minutes of arc exo fixation disparity in asymptomatic non-presbyopes.

Studying 105 subjects who were 8 to 71 years of age, Karania and Evans ( 1 ) found that those who had a fixation disparity on a standard Mallett unit had higher symptom scores than those with zero fixation disparity. In the present study, there were more subjects with exo fixation disparity than with eso fixation disparity ( 12 ). Both those with exo fixation disparity and those with eso fixation disparity had approximately equal representation in symptomatic and asymptomatic groups (Table 4). Twenty-four of the 49 with exo fixation disparity were in the symptomatic group and 5 of 12 with eso fixation disparity were in the symptomatic group. In contrast, for those with zero fixation disparity, only 1 of 39 was classified as being symptomatic. The fact that the symptomatic group averaged more exo Y-intercept and more BI X-intercept can be explained by the preponderance of subjects with exo fixation disparity. 

In the studies of Ogle et al.(13) Sheedy and Saladin, (7) Wick (8), Wildsoet and Cameron (28) and Yekta et al.(17) the most prevalent curve type was type I, which is similar to our results. The previous studies reported that curve type is an important indicator of decompensated heterophoria, (7, 13) with curve type I as a normal curve and other curve types being more common in symptomatic subjects. In line with that report, the present study found more non-type I curves among symptomatic subjects. In the present study, chi-square showed a significant difference in the distributions of curve type between the symptomatic and asymptomatic groups. In this sample with a much higher prevalence of exo dissociated phoria than of eso dissociated phoria, a more exo Y-intercept, a more BI X-intercept, and a steeper slope were found with the modified Mallett unit in symptomatic compared with asymptomatic university students as the study population. The asymptomatic group had proportionately many more subjects with zero fixation disparity than the symptomatic group. There were more subjects with exo fixation disparity than with eso fixation disparity leading to the more exo Y-intercept and more BI X-intercept in the symptomatic group. Using one standard deviation from the mean for asymptomatic subjects as an abnormal slope value suggest that slopes more negative than -0.22 minutes of arc per prism diopter would be abnormal with this particular device.
